# Complete mitochondrial genomes of Baikal oilfishes (Perciformes: Cottoidei), earth’s deepest-swimming freshwater fishes

**DOI:** 10.1080/23802359.2017.1398603

**Published:** 2017-11-07

**Authors:** Michael W. Sandel, Andres Aguilar, Kayla Fast, Stephen O’Brien, Alla Lapidus, David B. Allison, Veronika Teterina, Sergei Kirilchik

**Affiliations:** a Department of Biological and Environmental Sciences, The University of West Alabama, Livingston, AL, USA;; b Department of Biological Sciences, California State University Los Angeles, Los Angeles, CA, USA;; c Theodosius Dobzhansky Center for Genome Bioinformatics, St. Petersburg State University, St. Petersburg, Russia;; d Halmos College of Natural Sciences and Oceanography, Nova Southeastern University, Fort Lauderdale, FL, USA;; e Centre for Algorithmic Biotechnology, St. Petersburg State University, St. Petersburg, Russia;; f Department of Epidemiology and Biostatistics, School of Public Health, Indiana University, Bloomington, IN, USA;; g Limnological Institute of the Russian Academy of Sciences, Irkutsk, Russia

**Keywords:** *Comephorus baicalensis*, *Comephorus dybowskii*, Golomyanka, oilfish, mtDNA, mitochondrial genome

## Abstract

Sculpins are predominantly benthic sit-and-wait predators that inhabit marine and freshwaters of the Northern Hemisphere. In striking contrast to riverine relatives, sculpins endemic to Lake Baikal have diversified in both form and function, with multiple taxa having adaptations for pelagic and bathyal niches within the world’s deepest lake. Baikal Oilfishes (*Comephorus* spp.) represent a highly apomorphic taxon with unique skeletal morphology, soft anatomy, and reproductive ecology. Selection for novel behavior and life history may be evident in genes responsible for organismal energy balance, including those encoding subunits of the electron transport chain. Complete mitochondrial genomes were sequenced for the Big Baikal Oilfish (*Comephorus baicalensis*) and Little Baikal Oilfish (*Comephorus dybowskii*). Mitochondrial genomes encode genes essential for electron transport, and data provided here will complement ongoing investigations of genome-to-phenome maps for teleost respiration and metabolism. Phylogenetic analyses including oilfish mitogenomes and all publicly available cottoid representative sequences are largely concordant with previous studies.

## Introduction

The Big Baikal Oilfish (*Comephorus baicalensis;* Pallas 1776) and Little Baikal Oilfish (*Comephorus dybowskii*; Korotneff 1904) are known locally as Golomyanka, which is derived from the old Russian word “Golomen,” meaning “open sea, far from shore.” This corresponds well with the species’ pelagic habits within Lake Baikal, as oilfishes undergo diel migration to the bottom of the world’s only oxygenated bathyal (>1800 m) freshwater ecosystem (Sideleva 2003). Both species are distinguished from stream-dwelling relatives by extraordinarily high lipid content (≤40%), reduced dermal pigmentation and retinal development, elongated fins and skull bones, and viviparity. Like all freshwater sculpins, oilfishes lack a gas bladder, but high proportional body fat enables evolutionary specialization on a pelagic life history. Such adaptations may have facilitated oilfishes becoming the dominant vertebrate taxa within Lake Baikal, both in terms of biomass and abundance (Sideleva 2003; Teterina et al. 2010). Oilfishes support a substantial local fishery, and they comprise the primary food item of the Nerpa, a Baikal endemic seal. *Comephorus baicalensis* is distinguished from *C. dybowskii* by having greater total length, greater proportional orbit length, shorter proportional pectoral fins, and smaller cephalic pore chambers (Taliev 1955).

Life at great depth is subject to relatively uncommon selective forces, including extraordinary hydrostatic pressure, which may disrupt homeostasis. In species that utilize environments varying greatly in hydrostatic pressure, gene products must exhibit functional plasticity in order to maintain homeostasis. Considering that *Comephorus* species are known to migrate vertically through the Baikal water column, we hypothesize that the genome sequences of *Comephorus* species will reveal important information regarding adaptation to hydrostatic plasticity in vertebrates. In order to explore genome-to-phenome maps for Oilfish physiology and morphology, we present the first complete mitochondrial genome sequences for *C. baicalensis* and *C. dybowskii*.

## Data generation

Whole genomic DNA was isolated from fin clips collected from two specimens for each species, and voucher material was retained at the Sandel Laboratory of Aquatic Evolution at UWA. Mitochondrial genomes were generated using traditional Sanger sequencing at the Limnological Institute of the Russian Academy of Sciences and sequencing-by-synthesis on illumina HiSeq at the UAB Heflin Center for Genomic Sciences. Sanger reads were trimmed and aligned with Bioedit 7.0.0 (Hall 2005), and HiSeq reads were assembled using MITObim 1.7 (Hahn et al. 2013) on the CHEAHA central computing resource at the University of Alabama at Birmingham. The mitochondrial genome of *Cottus poecilopus* (GenBank accession EU332750) was used as a reference sequence. A multiple alignment was conducted with MAFFT version 7 (Katoh and Standley 2013) and validated by eye with Bioedit.

## Phylogenetic analysis

**Figure 1.**
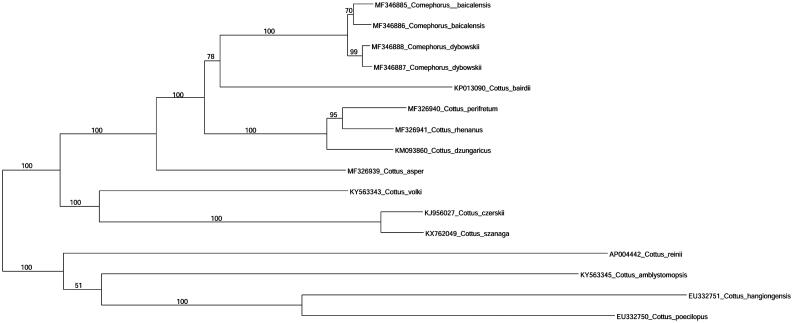
Interspecific phylogeny inferred under the maximum-likelihood (GTR + G + I) optimality criterion (Nei and Kumar 2000). Support values represent the proportion of 500 bootstrap replicates in which the associated taxa clustered together. Evolutionary analyses were conducted in MEGA6 (Tamura et al. 2013).

MEGA 6 was used to select the optimum nucleotide substitution model and conduct a maximum-likelihood phylogenetic analysis (Tamura et al. 2013; Figure 1). Minimum evolution and neighbour-joining trees resulted in the same tree topology as the maximum-likelihood tree. With the exception of the taxa added in this paper, phylogenetic analyses revealed the same tree topologies reported in recent studies of *Cottus* mitochondrial genomes (Balakirev et al. 2016; Han et al. 2016; Swanburg et al. 2016; Fast et al. 2017).

## References

[CIT0001] Balakirev ES, Saveliev PA, Ayala FJ. 2016 Complete mitochondrial genome of the Amur sculpin *Cottus szanaga* (Cottoidei: Cottidae). Mitochondrial DNA B Resour. 1:737–738. 10.1080/23802359.2016.1233470 PMC7871824 33644381

[CIT0003] Fast KM, Aguilar A, Nolte A, Sandel MW. 2017 Complete mitochondrial genomes for *Cottus asper*, *Cottus perifretum*, and *Cottus rhenanus* (Perciformes, Cottidae). Mitochondrial DNA Part B Resour. 2:666–668. 10.1080/23802359.2017.1375870 PMC7800474 33473940

[CIT0002] Hahn C, Bachmann L, Chevreux B. 2013 Reconstructing mitochondrial genomes directly from genomic next-generation sequencing reads-a baiting and iterative mapping approach. Nucleic Acids Res. 41:e129. 23661685 10.1093/nar/gkt371 PMC3711436

[CIT0004] Hall T. 2005. BioEdit version 7.0.0. Department of Microbiology, North Carolina State University.

[CIT0005] Han X, Li C, Zhao S, Xu C. 2016 The complete mitochondrial genome of Cherskii's sculpin (Cottus czerskii) (Scorpaeniformes: Cottidae)). Mitochondrial DNA A DNA Mapp Seq Anal. 27:2629–2630. 26024130 10.3109/19401736.2015.1041127

[CIT0006] Katoh K, Standley DM. 2013 MAFFT multiple sequence alignment software version 7: improvements in performance and usability. Mol Biol Evol. 30:772–780. 23329690 10.1093/molbev/mst010 PMC3603318

[CIT0007] Korotneff A. 1904 Résultats d'une expédition zoologique au Lac Baïkal pendant l'été de 1902. Arch Zool Exp Génér (Paris) Ser. 4. 2:1–26.

[CIT0008] Nei M, Kumar S. 2000 Molecular evolution and phylogenetics. New York: Oxford university press.

[CIT0009] Pallas PS. 1776. Reise durch verschiedene Provinzen des russischen Reiches. St. Petersburg. Volume 3. Voyages de m. P.S. Pallas, en différentes provinces de l'empire de Russie, et dans l'Asie septentrionale. Traduits de l'allemand par M. Gauthier de la Peyronie. [French translation].

[CIT0010] Sideleva VG. 2003 The endemic fishes of Lake Baikal. Leiden (The Netherlands): Backhuys; p. 270.

[CIT0011] Swanburg T, Horne JB, Baillie S, King SD, McBride MC, Mackley MP, Paterson IG, Bradbury IR, Bentzen P. 2016 Complete mitochondrial genomes for *Icelus spatula*, *Aspidophoroides olrikii* and *Leptoclinus maculatus*: Pan-Arctic marine fishes from Canadian waters. Mitochondrial DNA A DNA Mapp Seq Anal. 27:2982–2983. 26122337 10.3109/19401736.2015.1060472

[CIT0012] Taliev DN. 1955 The Sculpins of Lake Baikal (Cottoidei) [in Russian]. Academy of Sciences of the USSR. East Siberia Branch, Moscow; p. 603.

[CIT0013] Tamura K, Stecher G, Peterson D, Filipski A, Kumar S. 2013 MEGA6: molecular evolutionary genetics analysis version 6.0. Mol Biol Evol. 30:2725–2729. 24132122 10.1093/molbev/mst197 PMC3840312

[CIT0014] Teterina VI, Sukhanova LV, Kirilchik SV. 2010 Molecular divergence and speciation of Baikal oilfish (Comephoridae): facts and hypotheses. Mol Phylogenet Evol. 56:336–342. 20382246 10.1016/j.ympev.2010.04.001

